# Identifying Barriers and Facilitators to Providing Psychologically Safe Care in Inpatient Mental Healthcare. A Theoretical Domains Framework‐Informed Qualitative Study

**DOI:** 10.1111/jocn.70402

**Published:** 2026-06-15

**Authors:** Bethany Griffin, Chris Keyworth, Judith Johnson, John Baker

**Affiliations:** ^1^ School of Psychology University of Leeds Leeds UK; ^2^ School of Healthcare University of Leeds Leeds UK; ^3^ Division of Nursing, Midwifery and Social Work, Faculty of Biology, Medicine and Health, School of Health Sciences University of Manchester Manchester UK; ^4^ Manchester University NHS Foundation Trust Manchester UK; ^5^ School of Public Health and Community Medicine University of New South Wales Sydney New South Wales Australia

**Keywords:** inpatient, mental healthcare, nursing, psychological safety, restrictive practices

## Abstract

**Aim(s):**

To explore the facilitators and barriers to staff providing psychologically safe care in inpatient mental healthcare when restrictive practices are used.

**Design:**

Qualitative descriptive interview study.

**Methods:**

Twenty semi‐structured interviews were conducted with staff with experience working in inpatient mental healthcare in England. Analysis included principles of framework analysis, informed by the Theoretical Domains Framework.

**Results:**

Access to resources and a safe environment for both patients and staff were recognised as important (environmental context and resources) but access was impacted by competing organisational priorities and expectations (beliefs about capabilities). Participants recognised knowledge gaps in themselves and their colleagues (knowledge). Being able to confidently make decisions about risk was seen as central to the staff role (social/professional role and identity). Collaboration between staff is needed to make positive change and progression towards psychologically safe care (social influences). Empathy and compassion were driving factors in participants trying to use psychologically informed alternatives, but burnout hindered this (emotions).

**Conclusion:**

Ensuring that staff feel supported in their role to implement psychosocial informed alternatives to restrictive practices, as well as providing safe environments for both patients and staff, could support the integration of psychologically safe care on inpatient mental health wards.

**Implications for the Profession and/or Patient Care:**

Key facilitators and barriers to staff providing psychologically safe care are identified to support practice and improvements to patient care.

**Reporting Method:**

Consolidated criteria for reporting qualitative studies (COREQ).

**Patient or Public Contribution:**

Former patients and members of the public were involved in the conceptualisation of key concepts and design of this study.

## Highlights

**Impact:**

- Unsafe wards have an over‐reliance on restrictive practices, prioritising physical safety over the psychological safety of patients. This study is the first to explicitly explore how staff provide psychologically safe care in the context of restrictive practices, whilst also considering areas for organisational improvement.
- Staff recognise the importance of providing psychologically safe care but report the lack of support to do so. Key factors of healthcare practice both support and hinder this.
- Findings from this paper have implications for clinicians and organisations providing inpatient mental healthcare.

**What Does This Paper Contribute to the Wider Global Clinical Community?:**

- Ensuring that staff feel supported in their role to implement psychologically informed alternatives to restrictive practices, as well as providing safe environments for both patients and staff, could support the integration of psychologically safe care on inpatient mental health wards.
- This paper has identified key factors within mental health settings that limit opportunities for staff to provide psychologically safe care. It has also demonstrated how staff provide psychologically safe care within a restricted environment. Key learnings around organisational support could be transferred to other healthcare settings.

## 1 Introduction

Inpatient mental health wards provide care to people facing acute distress. In England, care can be provided on a voluntary or compulsory basis subject to assessment and detention under the Mental Health Act 1983 (amended as of 2025). All wards should follow a multi‐disciplinary approach to care comprising medical, nursing, allied health professionals, and psychological services (Health Services Safety Investigations Body [HSSIB] 2025). This approach allows for comprehensive and holistic treatment, under the assumption that multidisciplinary teams (MDTs) can improve the quality of care provided to patients (Haines et al. 2018).

There are concerns over the safety of people on inpatient mental health wards in England (Department of Health and Social Care 2024). Safety in this context refers to the protection from both physical and psychological harm, whilst considering risk, and sensory and support needs (Care Quality Commission 2024a). Unsafe wards have an overreliance on restrictive practices to contain perceived risk, prioritising physical safety over, and often at the expense of, psychological safety (Berzins et al. 2020; Care Quality Commission 2024b). Restrictive practices are deliberate acts that restrict movement, liberty and/or freedom to take control of a potentially dangerous or harmful situation (Department of Health 2014; National Institute for Health and Care Excellence [NICE] 2015); also referred to as coercion, coercive and restrictive interventions. Practices include physically intrusive methods such as restraint, seclusion, observations and rapid tranquilisation, and practices applied throughout a ward, like locked doors, surveillance and blanket restrictions (i.e., limitations on belongings and ward rules such as set mealtimes and bedtimes; NHS England 2025).

Restrictive practices have been shown to have detrimental impacts on patients, ranging from psychological harm (fear, distress and loss of trust) to physical harm (injuries and in some cases death; Chieze et al. 2019; Cusack et al. 2018). Despite this, restrictive practices are often described as necessary in times of crisis by patients and staff (Butterworth et al. 2022). It is possible that the psychological harm caused by restrictive practices could be minimised by supportive communication, patient involvement in decisions around their use and promoting positive therapeutic relationships through relational care (Griffin, Baker, et al. 2025).

## 2 Background

Recently, consideration of patients' psychological safety has been recognised as important for the wellbeing of patients in mental health settings and is included in the recent NHS Culture of Care Standards (NHS England 2024). Previous qualitative research has demonstrated times when patients felt psychologically safe and acknowledges that when communication is prioritised, psychological harm from restrictions can be minimised (Cutler et al. 2020; Griffin, Johnson, et al. 2025; Vogt et al. 2024). Research exploring what psychologically safe care looks like in practice is in its infancy and is limited to the patient perspective, meaning how staff might provide psychologically safe care has not yet been explored.

There are many challenges that mental health services in England are facing, including reports of insufficient funding, lack of training, support for staff, and staffing factors impacting patient care (British Medical Association 2024). As such, identifying these barriers in the context of staff providing high quality, psychologically safe care to patients is needed. Similarly, identifying times when good quality care is achieved whilst under constraints of limited resources, from the perspective of the people working in those settings, is important for moving forward to a more holistic view of safety on inpatient mental health wards.

The use of behavioural theories allows for underlying structural and psychological processes to be explored in relation to the implementation of new ways of working (Atkins et al. 2017). The Theoretical Domains Framework (TDF) was developed with the aim to identify influences on the behaviour of healthcare professionals (Cane et al. 2012). The fourteen‐domain TDF allows for the cognitive, affective, social, and environmental influences on healthcare professionals' behaviour to be explored to develop actionable recommendations (Atkins et al. 2017). The TDF has been extensively used to understand healthcare professional behaviours (Atkins et al. 2017), for example investigating the implementation of interventions in both general and mental healthcare settings (Chung et al. 2023; Johnston et al. 2022). While the framework has been used in mental healthcare settings, it has not been used to understand the barriers and facilitators to providing psychologically safe care in inpatient mental healthcare.

## 3 The Study

Exploring how staff provide psychologically safe care to patients, in the context of restrictive practices, is important for the development and implementation of future recommendations, as well as understanding how interventions could be developed and incorporated into existing organisational structures. The current study aimed to explore the facilitators and barriers to staff providing psychologically safe care in inpatient mental healthcare when restrictive practices are used to answer the research question, ‘How do staff in inpatient mental health care provide psychologically safe care to patients?’

## 4 Methods

### 4.1 Design

This study used a qualitative descriptive design using semi‐structured interviews and was informed by principles of the framework approach (Gale et al. 2013). The topic guide and analysis were underpinned by the 14 domains of the Theoretical Domains Framework (TDF) (Table 1).

**TABLE 1:** The theoretical domains framework (v2) with definitions and constructs (Atkins et al. 2017).

Domain	Definitions	Constructs
1. Knowledge	An awareness of the existence of something	Knowledge (including knowledge of condition/scientific rationale) Procedural knowledge Knowledge of task environment
2. Skills	An ability or proficiency acquired through practice	Skills Skills development Competence Ability Interpersonal skills Practice Skill assessment
3. Social/professional role and identity	A coherent set of behaviours and displayed personal qualities of an individual in a social or work setting	Professional identity Professional role Social identity Identity Professional boundaries Professional confidence Group identity Leadership Organisational commitment
4. Beliefs about capabilities	Acceptance of the truth, reality or validity about an ability, talent or facility that a person can put to constructive use	Self‐confidence Perceived competence Self‐efficacy Perceived behavioural control Beliefs Self‐esteem Empowerment Professional confidence
5. Optimism	The confidence that things will happen for the best or that desired goals will be attained	Optimism Pessimism Unrealistic optimism Identity
6. Beliefs about Consequences	Acceptance of the truth, reality, or validity about outcomes of a behaviour in a given situation	Beliefs Outcome expectancies Characteristics of outcome expectancies Anticipated regret Consequents
7. Reinforcement	Increasing the probability of a response by arranging a dependent relationship, or contingency, between the response and a given stimulus	Rewards (proximal/distal, valued/not valued, probable/improbable) Incentives Punishment Consequents Reinforcement Contingencies Sanctions
8. Intentions	A conscious decision to perform a behaviour or a resolve to act in a certain way	Stability of intentions Stages of change model Transtheoretical model and stages of change
9. Goals	Mental representations of outcomes or end states that an individual wants to achieve	Goals (distal/proximal) Goal priority Goal/target setting Goals (autonomous/controlled) Action planning Implementation intention
10. Memory, attention and decision processes	The ability to retain information, focus selectively on aspects of the environment and choose between two or more alternatives	Memory Attention Attention control Decision making Cognitive overload/tiredness
11. Environmental context and resources	Any circumstance of a person's situation or environment that discourages or encourages the development of skills and abilities, independence, social competence and adaptive behaviour	Environmental stressors Resources/material resources Organisational culture/climate Salient events/critical incidents Person × environment interaction Barriers and facilitators
12. Social influences	Those interpersonal processes that can cause individuals to change their thoughts, feelings, or behaviours	Social pressure Social norms Group conformity Social comparisons Group norms Social support Power Intergroup conflict Alienation Group identity Modelling
13. Emotion	A complex reaction pattern, involving experiential, behavioural, and physiological elements, by which the individual attempts to deal with a personally significant matter or event	Fear Anxiety Affect Stress Depression Positive/negative affect Burn‐out
14. Behavioural regulation	Anything aimed at managing or changing objectively observed or measured actions	Self‐monitoring Breaking habit Action planning

### 4.2 Study Setting and Recruitment

Participation was sought from a range of staff who had more than 1 year's experience of working on an inpatient mental health ward in England, with the aim of speaking to a range of staff working day‐to‐day as part of the Multi‐Disciplinary Team (MDT). Staff with experience only in forensic settings or who had only worked with children and adolescents were not eligible for participation. Participants had to be able to provide informed consent. The study recruited through social media (using volunteer sampling) instead of recruiting through NHS trusts, to allow participants to speak freely about their experiences at work with the understanding that their participation would not be known by their employer. Trusts are NHS organisations responsible for directly managing and providing healthcare services in England and Wales. Trusts are typically responsible for several hospitals and services within or across counties. Initially, participants were mainly mental health nurses (including ward managers and reducing restrictive practice leads); therefore, a decision was made to purposively sample healthcare assistants and psychologists to allow representation from several roles within the MDT. Snowball sampling was also used whereby agreeable recruited participants shared the advert within their networks.

### 4.3 Positioning the Research Team

The lead researcher is a female PhD student that has no prior experience working as, or being a patient in, inpatient mental healthcare. This work was undertaken as part of her PhD project. The supervision team has extensive experience working in (JB is a mental health nurse and JJ is a clinical psychologist) and researching inpatient mental healthcare and restrictive practices, and two members of the research team have expertise in the Theoretical Domains Framework (CK and JJ). Before the current study was designed and carried out, the researcher had conducted interviews for a separate study with patients and had started the analysis of their experience. The researcher acknowledged the potential impact this could have on interview questions. Members of the research team (JB and JJ) and two independent lay leaders reviewed the interview materials. Lay leaders are members of the public that advise on research projects. Both lay leaders had previous experience working in mental health care but now work with the Yorkshire and Humber Patient Safety Research Collaboration as lay leaders. It is also recognised that the researcher's positioning as an outsider (has not been a patient in, or worked in, a mental health setting) could have meant that there were fewer personal points of connection for the participants and the researcher when interpreting data. Similarly, this could mean that the researcher was less sensitive to the issues raised by the participants. However, the researcher was supervised by senior researchers with experience working in inpatient mental health services and this perspective could have led to deeper and more insightful questioning during the interviews to aid the researcher's understanding.

Researching a complex area like applied health services, requires a critical approach and a recognition of the individual experience in context. For this study, it was important to consider the wider mental healthcare context, organisational priorities and the participant's experience within this. As such, this study was informed by a critical realist paradigm, acknowledging that staff experiences are shaped by organisational conditions but interpreted through individual perceptions (Alderson 2021). Critical realism acknowledges the realities of the participant but considers that the experience of an individual does not exist in isolation and is because of relationships and interactions with external structures (i.e., ward environment, staff skills and policy) that have influenced those experiences and realities (Fletcher 2017).

### 4.4 Data Collection

The study poster was shared on X, LinkedIn and Facebook groups consisting of mental health staff and listed restrictive practices as follows: locked doors, seclusion, restraint, rapid tranquilisation, observations and limitations on movement (including segregation). Participants were given the option to participate through MS Teams or via a phone call. Interested participants emailed the lead researcher and were provided with an information leaflet and opportunity to ask any questions about the study before agreeing to take part. Consent was recorded via Qualtrics.

The topic guide was informed by the 14 domain TDF using guidance from a methodology paper (Atkins et al. 2017) and was developed in collaboration with the research team and two additional people who had previously worked in mental health. The questions centred around the TDF domains in the context of inpatient mental healthcare. The full topic guide can be found in Supporting Information S1. Interviews were carried out by the first author between September 2024 and February 2025. Participants were encouraged to discuss how they provided psychologically safe care at work in the context of any ward experience that they deemed restrictive. After the interview, participants were sent a debrief email containing a £30 voucher. Recruitment ended when a range of staff roles were included, and a range of restrictive practices had been discussed. Interviews were audio recorded and transcribed verbatim using MS Teams, and the transcripts were checked for accuracy.

#### 4.4.1 Consideration of the Relationship Between Researcher and Participants

To build rapport with participants in the current study, there was initial contact over email. Here, participants had the opportunity to ask questions, state preferences, and request the topic guide in advance. Before the interview began, the researcher re‐introduced themselves, stating that they were a PhD student with no prior experience as an inpatient or as staff in mental health services. Participants were reassured that the interviewer wanted to hear their story and were happy for the participant to discuss any experience of providing psychologically safe care, in the context of restrictive practices, that they felt comfortable with. While transcripts were not returned to participants after the interviews, participants did have the opportunity to view and comment on the results of the study prior to submission for peer review.

### 4.5 Data Analysis

Data was analysed using principles of the framework approach: transcription, familiarisation, coding, identification of the framework, charting and interpretation (Gale et al. 2013; Keyworth et al. 2019).

#### 4.5.1 Phase One: Familiarisation With the Data

The interviews and analysis were led by the lead researcher, meaning that familiarisation began during the interviews when contextual notes were made. The transcripts and audio recordings were reviewed, and the researcher's own thoughts, emotions and reflections were noted. On the second read through, additional notes of contextual information (participant role, work setting, views on restrictive practices and understanding of psychological safety) were made.

#### 4.5.2 Phase Two: Coding

Inductive coding was used; generated from the data following an unrestricted approach (Gale et al. 2013). This process was supported by the software NVivo. The coding was initially done independently and took a semantic approach starting with the first interview and going in chronological order. An iterative approach was taken whereby initial codes were revisited and developed through reflecting on the initial interpretation leading to deeper, latent coding. The aim of coding was to follow an open coding process whereby anything related to staff providing psychologically safe care to patients, or incidences where that could not be prioritised, was identified.

#### 4.5.3 Phase Three: Identification of the Framework

Coding was discussed in an analysis meeting. Underlying themes of ‘culture’, ‘attitudes’, ‘understanding’ and ‘requirements of the role’ were identified to have positive and negative influences on whether staff could provide psychologically safe care for patients. Following the initial inductive coding process, emerging codes and themes were deductively mapped onto the 14 domains of the TDF to organise and present the nuance and complexity of staff experiences of providing psychologically safe care in the context of restrictive practices.

#### 4.5.4 Phase Four: Charting

A matrix was developed by the lead researcher with the domain and explanations (following guidance; Atkins et al. 2017) and the charted codes. A researcher with experience with the TDF (CK) independently coded and charted 35% (approximately 60 quotes) of the quotes onto the TDF. Agreement was reached based on an iterative process between the researchers and any disagreements were presented to the research team. Two criteria were used to establish the importance of a TDF domain: assessing the number of times a domain was mentioned across participants and qualitatively assessing where a domain was explicitly labelled as a barrier or facilitator. This approach is consistent with previous studies (Keyworth et al. 2019).

#### 4.5.5 Phase Five: Interpretation

The TDF allowed for the identification of relevant behavioural domains but further interpretation of how the domains impacted whether psychologically safe care was delivered was needed. As such, the codes within each domain were then further analysed into explanatory themes to aid interpretation of the facilitators and barriers associated with each domain of the TDF. This followed an iterative process, revisiting the quotes and developing the codes to eventually develop explanatory themes. This process was led by the lead researcher and codes and themes were checked during ongoing research meetings with the team until the explanatory themes were developed. The detailed analytical process, progressing from inductive coding to deductive mapping and thematic interpretation demonstrates how the facilitators and barriers were developed from the initial interview transcripts.

Throughout the analysis process, rigour was ensured through several mechanisms. First, regular meetings with the research team were held to debrief on interpretation. Second, independent coding and charting of 35% of the quotes onto the TDF framework by a second coder whereby discrepancies were reviewed and resolved through discussion. Third, reflexive notes were kept by the lead researcher throughout data collection and analysis to support transparency of interpretation of the presented discussion points. The regular research team meetings allowed for any areas of contention to be discussed and led to codes and themes being refined accordingly. This process, alongside ongoing critical reflection during the drafting of the manuscript, contributed to the trustworthiness of the analysis.

### 4.6 Ethical Considerations

A favourable ethical review was received by the University of Leeds, School of Psychology Ethics Committee (07/09/2024; reference number: PSCETHS‐1127). A £30 voucher was offered to participants to thank them for taking part. This amount was approved by the ethics committee.

## 5 Findings

There were 20 participants with a mean age of 37.4 years (range: 23–60 years). Participants had a range of job roles: nurse (*n* = 7; including ward managers and reducing restrictive practice leads), clinical psychologist (*n* = 5), assistant psychologist (*n* = 2), pharmacist (*n* = 2), healthcare assistant (*n* = 2), peer‐support worker (*n* = 1) and occupational therapist (*n* = 1). They were predominantly White British (*n* = 17); one participant was from a mixed ethnic background (Black Caribbean and White British), one participant was White Irish, and one participant did not disclose their ethnicity. See Table 2 for demographic information, including the restrictive practices discussed. MS Teams was used for 19 of the interviews, with one person participating via phone call. Interviews ranged from 31 to 65 min (mean 53 min). Total data amounted to 17 h and 21 min.

**TABLE 2:** Participant demographics and quote identification.

Participant ID	Gender	Age range (Years)	Occupation discussed	Inpatient setting	Tenure in mental healthcare (Years)	Region of UK worked in	Restrictive practices discussed
Participant 1	Male	46–55	Mental health nurse	Eating disorder wards	16	West Midlands	Locked doors, observations, physical restraint
Participant 2	Female	26–35	Occupational therapist	Adult acute wards	16	North West	Seclusion, segregation, blanket restrictions
Participant 3	Female	36–45	Deputy ward manager (mental health nurse)	Female adult acute ward	3	North West	Blanket restrictions
Participant 4	Male	46–55	Pharmacist	PICU and adult acute wards	25+	North West	Rapid tranquillisation
Participant 5	Female	26–35	Reducing restrictive practices nurse	Trust wide	12	North West	Seclusion, restraint, observations and blanket restrictions
Participant 6	Female	36–45	Bank healthcare assistant	PICU and adult acute wards	2	North East	Restraint, observations
Participant 7	Female	26–35	Ward manager	Adult acute ward	15	South West	Seclusion, nasogastric feeding under restraint, intramuscular injection under restraint
Participant 8	Male	46–55	Ward manager	Adult acute ward	30+	South West	Seclusion, segregation
Participant 9	Female	26–35	Clinical lead	Acute assessment unit	9	North East	Seclusion, restraint
Participant 10	Female	46–55	Pharmacist	PICU	20	South West	Rapid tranquillisation
Participant 11	Female	26–35	Ward manager	PICU	10	North West	Restraint, seclusion, blanket restrictions
Participant 12	Female	26–35	Healthcare assistant	Eating disorder unit and personality disorder unit	2	North West	Observations, seclusion, restraint, nasogastric feeding under restraint and locked doors
Participant 13	Female	18–25	Peer Support Worker/Expert by experience	Disordered eating ward and personality disorder ward	1	North West	Blanket restrictions, nasogastric feeding under restraint, locked doors
Participant 14	Female	18–25	Assistant psychologist	Mixed gender adult acute ward	1	North West	Seclusion, coercive language, observations
Participant 15	Female	26–35	Clinical psychologist	Two adult acute wards (one male, one female)	2	North West	Locked doors, blanket restrictions (meal times, standard menu and limit on phone chargers)
Participant 16	Male	26–35	Principal clinical psychologist	Adult inpatient wards	3	North West	Blanket restrictions
Participant 17	Female	26–35	Assistant psychologist	Previously adult acute ward, current older adult acute ward	5	North West	Blanket restrictions, restraint, seclusion, rapid tranquillisation
Participant 18	Female	26–35	Principal clinical psychologist	Two adult acute wards (one male, one female)	3	North West	Restraint, seclusion, rapid tranquillisation
Participant 19	Female	26–35	Clinical psychologist	Two adult acute wards (one male, one female)	2	North West	Locked wards, restraint, blanket rules
Participant 20	Male	56–65	Principal clinical psychologist	Trust wide (acute wards and PICU)	25	North West	Restraint, seclusion, psychological restraint

Six of the 14 TDF domains were identified as particularly salient within the analysis, each with associated explanatory themes. The most salient TDF domains were: environmental context and resources (138 occurrences, reported by 20 participants), beliefs about capabilities (62 occurrences, reported by 17 participants), knowledge (48 occurrences, reported by 12 participants), social/professional role and identity (42 occurrences, reported by 15 participants), social influences (40 occurrences, reported by 13 participants), and emotions (39 occurrences, reported by 12 participants). Quotes are included to provide context in the participants' own words. Additional quotes are provided in Supporting Information S2.

### 5.1 TDF Domain 1: Environmental Context and Resources

Access to resources and a safe environment for both patients and staff were recognised as imperative for the psychological safety of patients. The ward environment and negative cultures hinder proactive and psychologically informed ways of working. Two facilitators and three barriers were identified.

#### 5.1.1 Facilitators

##### 5.1.1.1 Resource

Participants described the importance of resources including psychologically informed alternatives to risk assessment, patient preferences and history, adequate staffing levels without reliance on agency staff, and wellbeing support for staff. Some participants mentioned that having access to these resources on the ward often led to a less risk‐focused and restrictive form of care. Staffing challenges were mentioned by most participants, with the belief that if their ward had access to adequate staffing, restrictive practices would be reduced.

‘I mean it always comes back to sort of resources and staffing. I think because things are so stretched, people aren't able to think psychologically or think holistically. They are just focused on risk.’ Clinical Psychologist (Participant 15)

##### 5.1.1.2 Creating Safe Spaces for Patients

Participants discussed the importance of patients having access to a safe space, for example a private bedroom that they felt comfortable in. Where a safe environment for patients was absent, this was created by staff through holding meetings away from bedrooms, using ‘knock and wait’ and consistently trying to meet patient preference, even when staff did not think the preference would be helpful for patients. Accepting this conflict was seen as part of the role.

‘We work psychologically within the limitations of that restriction … we have 11 different groups or activities that we facilitate each week to try and continue to promote that psychological safety on the ward, even with the restrictions’. Assistant Psychologist (Participant 17)

#### 5.1.2 Barriers

##### 5.1.2.1 Ward Environment

All participants mentioned their work environment, described as both the physical structures (i.e., ward layout and the condition of communal spaces) and patient‐mix (i.e., patient acuity and interactions of patients on the ward), when discussing psychologically safe care. Participants described the impact that the environment can have on a patient's thoughts and emotions and in turn the behaviour that follows, such as the impact of a busy area of the ward. Some staff discussed how their response to a patient's behaviour is dependent on how ‘acute’ the ward is, for example higher acuity can result in more restrictions and less use of psychologically informed alternatives. Providing psychologically safe care in their current workplace felt impossible for some and for others an aspiration to work towards. To some, the ward environment needs the addition of restrictive practices and ward rules to keep patients safe.

‘You would think that by having no restrictive practices that would enhance the psychological safety on the ward. But I guess when working in healthcare there are various different things, which mean that the two have to go hand in hand and sit next to each other and complement each other.’ Occupational Therapist (Participant 2)

##### 5.1.2.2 Lack of Time

Nurses described not having the time to implement psychologically informed ways of working on top of their responsibilities. Responding to incidents, medication rounds, and ensuring the physical safety of people on the wards were viewed as a priority. Working in a fast‐paced environment meant that some participants felt themselves and their colleagues did not have time to reflect on their decisions or the incidents they had witnessed on shift.

‘I think that that's what that boils down to is time on your shift. You work a 12 1/2 hour shift and you get 9 hours into it and you think, oh my God, the list of things I've got to do is like this long still.’ Nurse (Participant 7)

‘Well, it's just constantly chasing your tail because as soon as you resolve one thing, the other thing pops up and it's just the actual definition of madness.’ Clinical Psychologist (Participant 18)

##### 5.1.2.3 Staff Culture

Many participants perceived a negative work culture and identified the role management plays in creating this. Participants felt that poor staff culture translated into patients receiving poor quality care. A lack of support for non‐nursing staff was mentioned.

‘It's down to management, down to what staff are in that day. You knew what the day would be like. I think you even knew how many kickoffs [violent incidents] there would be by the staff you saw in the morning because are these people going to talk people down or are they quite kind of trigger happy not having any nonsense from anyone.’ Health Care Assistant (Participant 6)

### 5.2 TDF Domain 2: Beliefs About Capabilities

When staff felt confident in their assessment of risk and ability to face complexity in patient presentation, they were more likely to implement psychologically safe alternatives. However, this was impacted by organisational priorities and expectations. Two facilitators and two barriers were identified.

#### 5.2.1 Facilitators

##### 5.2.1.1 Importance of Understanding Actual Risk

Being able to accurately assess risk through proactive, meaningful conversations with patients around restrictive practices was a driving factor in whether restrictive practices were used or not. Staff that understood a patient's presentation and associated risk felt they could implement psychologically informed alternatives and have honest conversations with patients about how that impacts their decision making.

‘I will honestly say to a patient you are worrying me like I feel really threatened right now and then they'll say back, well, I don't mean to be threatening, and sometimes it just, you know, opens that conversation of the threat and the risk that we see really … I suppose people could quite quickly move to IM [intra‐muscular] injection but actually you just need to create opportunities to see green behaviours from patients.’ Nurse (Participant 11)

##### 5.2.1.2 Dealing With Complexity

Feeling capable (described by participants as having self‐confidence and belief) to deal with the challenge of verbally communicating with unwell patients on busy wards with a mixture of patient needs was seen as imperative to the role for some participants. Facing complexity and finding alternative ways of working meant that participants could prioritise psychologically safe ways of working over restrictive practices.

‘I think maybe when those risks are higher, we go to sort of the medical model and medication and the kind of restriction rather than thinking about what's going on for people because it's too much to actually, sit with that and find that out would be so much more complex and there's something about, I think people struggle to sit with the complexity.’ Clinical Psychologist (Participant 15)

#### 5.2.2 Barriers

##### 5.2.2.1 Expectations vs. Reality

Some participants described the conflict between expectations and reality in the current mental health service climate with regards to providing psychologically safe care. Participants reflected that some decisions they make to meet organisational priorities and demands might not be patient centred and could compromise quality of care.

‘I think that it's not through a lack of staff really wanting and that is such a difficult thing in this job … there's things in the system and societally, and also in the immediate system of the [NHS] trusts and XY and Z that just we can't get there and that is suffocating to staff because all they want to do is to provide good quality care, which good quality care is psychological safety.’ Clinical Psychologist (Participant 18)

##### 5.2.2.2 Conflicting Priorities Limiting the Possibility of Psychologically Safe Care

Some participants recognised how the process‐driven nature of healthcare organisations does not allow for therapeutic, person‐centred opportunities. An organisational focus on patient flow, bed numbers, and external pressure and fear of litigation was mentioned. Some participants described how they felt that these priorities differed from patients' needs.

‘My gut reaction is, no management and leadership do not prioritise psychological safety. It's that physical safety that's the priority’. Pharmacist (Participant 4)

### 5.3 TDF Domain 3: Knowledge

One facilitator and one barrier were identified in relation to the knowledge domain. Knowing the patients that staff are caring for improved the care they provided. Knowledge gaps and the impact they can have on patient care were discussed.

#### 5.3.1 Facilitators

##### 5.3.1.1 Healthcare Professional—Patient Relationship

Knowing the patients meant that participants felt they could have conversations around the use of restrictive practices, patient preference and risk assessment. Some participants felt this improved the therapeutic relationship and allowed for an understanding of patient behaviour that mitigated the need for restrictive practices. Having awareness of psychologically informed alternatives supported this. Limiting the use of restrictive practices at work was seen as providing psychologically safe care by most participants.

‘If you'd been able to build up a relationship with somebody so that you could negotiate with them, but in a way that was really collaborative, I imagine that would help people to feel you're moving away from restriction and towards cooperation.’ Clinical Psychologist (Participant 19)

#### 5.3.2 Barrier

##### 5.3.2.1 Knowledge Gaps

Most participants recognised a lack of understanding of patient behaviour and disorders across the MDT. Concerns over lack of training and understanding for non‐nursing staff that had regular patient contact were expressed by some participants.

‘Like healthcare assistants, they don't have enough training to be dealing with people in such high stress environments.’ Peer Support Worker (Participant 13)

### 5.4 TDF Domain 4: Social/Professional Role and Identity

Two facilitators and one barrier were identified for this domain. Being able to confidently make and communicate decisions to patients was seen as central to the role. Inconsistency and staff concern over connections with patients were identified as barriers.

#### 5.4.1 Facilitators

##### 5.4.1.1 Professional Confidence to Make and Explain Decisions

Being able to assess risk confidently, work in alternative ways (e.g., symbol‐based communication) and make decisions effectively was seen as a key asset to the nursing role. Some participants also recognised the importance of having the confidence to question potentially detrimental practices by colleagues to ensure a positive culture, such as restraint being used without de‐escalation or questioning why someone is being moved to seclusion.

‘If I see something that I don't like, I will discuss it and it might just be that that's me, that doesn't like it and actually, that's the way it has to happen. And I'm quite happy to have that open conversation with people.’ Nurse (Participant 7)

##### 5.4.1.2 Building Therapeutic Relationships Through Engagement and Purposeful Communication

Ensuring that patients felt heard, recognising the importance of explaining decisions and having positive interactions and communication with patients daily were some of the ways that participants felt staff could add value to interactions on the ward. Some participants described how certain staff, such as named nurses, key workers and psychologists, were purposefully not involved in restrictive practices (i.e., restraint and rapid tranquilisation) to protect their relationship with the patients. Some participants described intentionally finding pockets of opportunity to engage with patients, for example during enhanced observations and informal communication in communal spaces.

‘Communication before, during and after … I have seen where people have been in restraint for minimal periods of time due to the way that staff have communicated with them … it just calms things, right down, and then everyone's fine.’ Healthcare Assistant (Participant 6)

#### 5.4.2 Barriers

##### 5.4.2.1 Inconsistency in Ways of Working

Participants perceived inconsistent work practices in mental health settings, particularly in the approaches of different staff on shift. Lack of definitions (e.g., trauma‐informed care and psychological safety) and standardised ways of working can mean that staff work in different ways, leading to conflicting decisions and messages to patients. Skill differences between different groups of staff, i.e., day and night staff and permanent and agency staff was briefly mentioned.

‘Some staff will encourage patients to sort of learn these therapy skills they've used, advocate for them when they can't and then sometimes you have others and they have absolutely no idea what's going on and they have no idea how to implement it and they say completely the wrong thing and make situations 10 times worse … it really just depends.’ Healthcare Assistant (Participant 12)

### 5.5 TDF Domain 5: Social Influences

The social relationships between staff, including having shared values and collaboration within a team, were seen as important to make positive change and progression towards safe care. Resistance to implement new ways of working and issues around engagement with patients were mentioned. One facilitator and two barriers were identified within this domain.

#### 5.5.1 Facilitators

##### 5.5.1.1 Collaboration and Shared Priorities

Staff buy‐in and collaboration were recognised as important for new ways of working to be adopted into day‐to‐day practice. Participants believed that knowledge sharing and a shared team focus is how good quality care, where alternatives to restrictive practices are considered, is achieved.

‘Everyone's embracing a new way of working, more therapeutic, a less restrictive way of working … it feels quite unified that everyone wants to progress and make it less restrictive.’ Nurse (Participant 8)

#### 5.5.2 Barriers

##### 5.5.2.1 Staff vs. Patients: Preventing Engagement With Care

Personal opinions, for example patients' views of staff and vice versa, were mentioned as preventing psychologically informed alternatives being used. Some participants described how themselves and their colleagues held certain beliefs about patient behaviour that meant they were less likely to engage with patients. Examples of these beliefs included: patients want attention and that unwell patients should take responsibility for behaviours that challenge.

‘I think there are some circumstances where patients simply won't allow you to do it in a psychologically safe way. There are some people that are so pissed off, so pumped up that actually no matter what you say you're not going to be able to do anything without restraint and without seclusion.’ Pharmacist (Participant 4)

##### 5.5.2.2 Resistance to Change

Concerns over multi‐disciplinary ways of working were reported by several participants, specifically the impact that more senior members of staff have on the direction of the teams' decisions. Clinical psychologists mentioned the notion of ‘battling against the MDT’ when consultants and nurses focused on delivering care according to the medical model in cases of trauma. Some participants felt that the implementation of psychologically informed ways of working was prevented because of resistance from their colleagues.

‘Obviously we've got matrons and senior leaders and people that will ask and see incident reports on the PICU and say, well, he needs this [restrictive practice] … I suppose they don't know the patient. They just see an incident, don't they?’ Nurse (Participant 11)

### 5.6 TDF Domain 6: Emotions

Empathy and compassion were driving factors in participants trying to use psychologically informed alternatives with patients. Staffs' emotional capabilities and burnout was seen to impact care. One facilitator and two barriers were recognised within this domain.

#### 5.6.1 Facilitator

##### 5.6.1.1 Empathy and Compassion

Having empathy and compassion for patients positively impacted the way staff provided care and communicated with patients. Some described how the context that decisions are made in, for example on a mental health ward compared to a public setting, impacts the acceptability of them. Participants discussed how they continued to communicate with people in crisis as they would with people not in crisis (e.g., not infantilising or patronising adult patients).

‘You can still make choices even if you are in a mental health crisis. Why do these people not get treated like adults. What is the problem here? I genuinely don't understand what the problem is, it's bizarre isn't it.’ Nurse (Participant 1)

#### 5.6.2 Barriers

##### 5.6.2.1 Burnout and Compassion Fatigue

Staff burnout and compassion fatigue limited staff's capacity to choose psychologically informed alternatives to restrictive practices, with one participant stating that the psychological safety of patients was “*the first thing to go in order to get through the shift*” ***Clinical Psychologist (Participant 18)***. Some participants recognised aspects of the jobs that contributed to stress and eventually burnout, for example, repeated incidents on a shift and staffing issues.

‘I think there's a massive link with burnout and stress and frustration and how it like impacts care … I suppose if you are feeling stressed and burnt out your tolerance isn't the same as that‐ your resilience isn't the same.’ Nurse (Participant 11)

##### 5.6.2.2 Fear and Personal Opinions Driving Decisions About Patient Care

Participants that feared for their own safety or being concerned for the repercussions if they were to not use restrictions meant that reactive care was more likely to be used. Concerns over damaged reputations because of incidents (e.g., patient suicide) was also described by some participants. Some participants said their colleagues used restrictive practices on patients they did not like or in some cases stereotyped certain diagnoses.

‘I think fear is always a big one. People are afraid of being hurt, and rightly so. You know, you're looking at a group of people who, on a day‐to‐day basis for very long days, very long hours, are exposed to significantly unpredictable unstable environments in which they receive an awful lot of abuse physically and mentally. And that has a price.’ Clinical Psychologist (Participant 20)

## 6 Discussion

This study is the first to identify facilitators and barriers to providing psychologically safe care in inpatient mental healthcare, from the perspective of healthcare staff. Two important contributions to the literature are made through this study. First, six prominent TDF domains were identified that participants felt were important in aiding or hindering their ability to support the psychological safety of patients. Secondly, staff recognise the importance of providing psychologically safe care but report the lack of support to do so in practice. Three key findings are discussed in relation to key components of mental health staff's' practice.

All participants recognised the ward environment as having the potential to both support and prevent staff from providing psychologically safe care to patients. Having access to resource and being able to modify the environment to support patients was seen as important. When there were factors that staff felt were out of their control (i.e., patient mix and organisational priorities), more restrictive measures were used. Staff have previously reported patient acuity and distress as barriers to the implementation of psychological therapies and how staff feel they lack control over ways of working set by organisations (Evlat et al. 2021; Polacek et al. 2015). The current study demonstrates that staff try to work within the restrictions to provide safe spaces for patients. Having access to resources is important in supporting this. The resources identified in the current study included, psychologically informed policies and guidelines, wellbeing support for staff and staffing resource. Healthcare providers, are facing resource and staffing issues, creating lack of time for meaningful engagement which is well‐documented in the UK literature (Evlat et al. 2021). While increasing resource and investment in psychologically safe alternatives should be priority for organisations, in the interim reporting and sharing the way in which staff are providing care to patients within constraints is important for shared learning across organisations.

Approaches to risk assessment and the management of self‐harm was discussed at length. Specifically, moving away from traditional and structured ways of assessing risk, such as structured questions and assumptions based on hospital records from previous admissions. Instead, staff in the current study described the importance of being proactive in having meaningful conversations with patients about their behaviours, instead of relying on structured questions often described as ‘tick‐box’ questions. Staff recognise the role that training plays in this to be able to confidently have these conversations instead of defaulting to risk‐averse, restrictive practices. The National Institute for Health and Care Excellence do not recommend traditional, ‘tick‐box’ risk assessment for self‐harm behaviours and instead support psychosocial assessment as a best practice preventative strategy (Guideline 225; National Institute for Health and Care Excellence [NICE] 2022). Psychosocial assessment encompasses a co‐produced formulation of a patient's needs, safety considerations and vulnerabilities to develop a shared understanding of a patient's behaviour that goes beyond quantifying risk (National Institute for Health and Care Excellence [NICE] 2022). Patients have expressed concerns over how traditional risk assessment is typically carried out, minimising the patient voice and replacing meaningful engagement with ‘tick‐box’ exercises (Deering et al. 2022). In the current study there was positive discussion about using risk assessment at work, but what this meant to the participants varied from traditional risk assessment to more formulaic assessments of risk. Given that their still seems to be reliance on ‘tick‐box’ methods of risk assessment, moving to more formulaic psychosocial assessments could be challenging. Interventions that focus on strengthening staff capability and capacity to carry out psychosocial risk assessment and providing skills‐based training could support this shift. Supporting staff to have meaningful conversations, where co‐produced formulations are developed is important for the future of management of aggression and self‐harm. The findings of this study support this shift in perspective in that participants accepted that this is an area of improvement needed on mental health wards.

Investment in staff knowledge and feelings about the use of restrictive practices at work was recognised as important by most participants. The current mental health workforce in England is facing challenges which could lead to dissatisfaction at work, burnout and compassion fatigue (Marshman et al. 2022). Burnout refers to emotional exhaustion, depersonalisation, disengagement and low personal accomplishment at work and is thought to be more prevalent in mental healthcare workers than staff in other settings (Johnson et al. 2018). Compassion fatigue refers to a loss of compassion in clinical practice due to the high demand of providing care in emotionally charged environments and can lead to lower quality care for patients due to reduced engagement (Marshman et al. 2022). Feeling valued, supported and psychologically safe in their role could potentially increase staff buy‐in to psychologically informed ways of working and positively impact the care patients receive. The link between psychological safety, burnout and patient outcomes is under‐researched (Montgomery et al. 2025) but has been identified in the current study as a concerning possibility if staff do not feel invested in and supported. The findings of this study demonstrate key areas of healthcare practice that could be targeted by tailored‐training packages to empower staff to provide care in line with their values. Examples include psychologically informed alternatives to restrictive practices, how to conduct co‐produced safety planning and building confidence in initiating complex, emotionally charged conversations about risk.

### 6.1 Implications for Practice and Research

The use of the TDF allowed for the identification of salient domains relevant to mental healthcare practice that can be targeted by organisations to implement psychologically safe care. Three specific recommendations for practice are outlined here. First, staff recognise the importance of psychological safety alongside physical safety but often feel it is not recognised by organisations, limiting their beliefs in their capabilities. Organisational buy‐in of psychologically informed training (e.g., human rights act training, de‐escalation and trauma related topics) and resource could drive cultural change, improving nurses' and healthcare assistants knowledge and morale to provide and engage meaningfully with patients. Second, collaboration between and within services is imperative for future improvements in patient safety at a national level. At present, staff recognise the fragmented and inconsistent ways of working between and within organisations, from undefined concepts and ideas and the use of different technologies and communication systems. An inconsistent environment for nurses and the wider MDT potentially translates to an inconsistent environment for patients. Third, meaningful engagement with patients should be a priority for nursing staff. Finding pockets of opportunities for engagement with patients, whether through observations, ward rounds or informal communication on the wards was recognised as important and feasible in the current study, despite working in restrictive environments. Providing and encouraging nursing staff to participate in ongoing reflective practice (where patient‐facing staff are provided time and supervision to reflect on their role and the decisions that they make at work) could facilitate these conversations with patients and avoid compassion fatigue.

There are two recommendations for future research based on the findings from this study. First, future research should focus on identifying ways in which psychologically safe care can be provided in the current mental health services climate, including combining suggestions from nursing staff, the wider MDT and patients to ensure any recommendations are grounded in the voice of the people experiencing these environments. It is imperative that any future interventions, whether that be for reducing restrictive practices, implementing trauma‐informed care or workforce initiatives, are acceptable and feasible for the staff and organisations implementing them. Acceptability (how people think and feel about interventions; Sekhon et al. 2017) has been identified as a key driver to intervention effectiveness and implementation (Skivington et al. 2021), and participants in the current study recognised the importance of belief in their capabilities to drive change. Further exploration of how these barriers can be addressed prior to intervention development is needed. Second, this study was conducted in England and captures experiences shaped by England‐centric policy and law, for example the Mental Health Act. It is possible that the identified domains and several findings might align with international priorities and barriers to change as reducing restrictive practices and integrating psychologically informed alternatives is a global priority (World Health Organization 2023). As such, countries facing similar healthcare pressures or that use similar MDT structures may resonate with the findings of the current study, but future research could build on the current findings to explore how psychologically safe care could be provided within different service delivery contexts across the UK and internationally.

### 6.2 Strengths and Limitations of the Work

The current study is the first to use the theoretically grounded TDF to identify facilitators and barriers for staff to provide psychologically safe care, demonstrating how staff recognise the importance of supporting the psychological safety of patients alongside protecting and maintaining their physical safety. There are four key limitations to note. The first limitation to note is around the sampling and resulting participants. This study only recruited participants that worked in England and as the participants shared the study within their teams, several participants were from the same NHS trusts and could have potentially shared similar experiences that could impact the results. Second, participants were primarily white and female staff. While recruitment was focused on discussing a range of restrictive practices and the inclusion of a range of healthcare professionals, the demographics of participants are not entirely representative of the NHS mental healthcare workforce and should be interpreted with this in mind (GOV.UK 2023). Third, while the study incorporated a wide range of job roles representing the MDT, there were key roles missing such as psychiatrists and social workers. Fourth, while the term restrictive practices is used in this study, methods such as technological surveillance and cultural restraint that are included in the recent NHS England guidance (NHS England 2025) were not included in this study. It is important that researchers, clinicians and policy makers are consistent in language and terminology used in this area.

## 7 Conclusions

The current study identifies key facilitators and barriers to staff in inpatient mental health settings providing psychologically safe care. The use of the TDF allowed for the identification of salient TDF domains that organisations could target to support the integration of psychologically safe care into practice. Ensuring that staff feel supported in their role to implement psychologically informed alternatives to restrictive practices, as well as providing safe environments for both patients and staff could support the integration of psychologically safe care on inpatient mental health wards. Supporting the psychological safety of patients whilst protecting them from physical harm is recognised as integral to caring for patients in acute distress.

## Author Contributions

The authors confirm their contribution to the paper as follows. Conceptualisation and Design: Bethany Griffin, John Baker and Judith Johnson. Development of Research Materials: Bethany Griffin, John Baker and Judith Johnson. Data Collection: Bethany Griffin. Analysis: Bethany Griffin, supported by Chris Keyworth. Draft Manuscript Preparation: led by Bethany Griffin. All authors reviewed the results and approved the final version of the manuscript.

## Funding

This work was supported by NIHR Yorkshire and Humber Patient Safety Translational Research Centre (Grant PSTRC2016‐006).

## Conflicts of Interest

The authors declare no conflicts of interest.

## Supporting information


**Supporting Information: S1.** Topic guide.
**Supporting Information: S2** Additional quotes to provide support to the explanatory themes.

## Data Availability

Research data are not shared.
