# Supplementary material for: Identifying Barriers and Facilitators to Providing Psychologically Safe Care in Inpatient Mental Healthcare. A Theoretical Domains Framework‐Informed Qualitative Study
Source: J Clin Nurs. 2026 Jun 15;35(11):4991–5005. doi: 10.1111/jocn.70402 (PMC13629262; doi:10.1111/jocn.70402)
Supplement: Supplementary file 1 [file JOCN-35-4991-s001.docx]

**Supplementary Material**

S1. Topic guide

S2. Additional quotes to provide support to the explanatory themes

**S1. Topic guide**

*Defining psychological safety*

While psychological safety is a recognised concept in mental healthcare, to the author’s knowledge, there is no clear, widely recognised definition in literature or practice. As such, a working definition was devised for the study informed by literature, interviews with former inpatients and input from a lived experience advisory group (LEAG): “*Feeling validated in your experience of the world and the belief you will be treated fairly based on your individual needs. Being psychologically safe provides protection from lasting psychological harm from your environment. It’s not just about being physically safe but being protected from events that may have lasting effects in the future.*”

**Introduction and definition**

- Can you tell me a little bit about yourself and your role in inpatient mental health please?
  - Cover what role they are in, how long they have been in the role and what setting.
  - Use of restrictive practices at work and their opinion on this.
  - Previous experience, motivations for working in this area.
- We have asked former patients what psychological safety means to them; this is what they said: “*Psychological safety is feeling validated in your experience of the world and the belief you will be treated fairly based on your individual needs. Being psychologically safe provides protection from lasting psychological harm from your environment. It’s not just about being physically safe but being protected from events that may have lasting effects in the future.”*
  - Is psychological safety a term you have heard before taking part in this study?
  - Can you tell me what your general views are of this definition?
  - Do you agree with the definition?

**Incorporating psychological safety into their role**

- Thinking about yourself at work, have there been times in the past where you have tried to help patients feel more psychologically safe?
  - If so, how did you do this?
  - If not, why not?
- Is increasing psychological safety a priority on your ward?
  - If not (and they themselves have done it) where did you get the idea to work in this way?
  - If yes, how are you encouraged to do this? What resources are you provided with?

**Using restrictive practices on the ward**

- How do you think providing psychologically safe care aligns with the use of restrictive practices?
  - Do you think that the use of restrictive practices on the wards are always necessary? If yes/no, why?
- Thinking about a time when you have used restrictive practices on the ward, how did you incorporate providing psychologically safe care during this time?
  - If they didn’t – on reflection, how could you have promoted the psychological safety of patients during that incident?
- Are you able to work in this way every time you use **X restrictive practice?**
  - Is there anything that hinders your ability to work in this way? – prompts: emotions of patients and staff, support by the organisation.
  - Do you think there are any particular skills needed to work in this way? Is this something you feel you can do well?
  - Do the people you work with prioritise the psychological safety of patients?

**Alternatives and suggestions**

- If you could speak to your trust’s leadership and policy makers, what suggestions would you give for making restrictive practice more psychologically safe in inpatient mental healthcare?
  - What could be implemented or changed on the ward to reduce the psychological harm of restrictive practice or make it unnecessary?

**S2. Additional quotes to provide support to the explanatory themes**

| **Domain** | **Explanatory Theme** | **Example quotes** |
| --- | --- | --- |
| Environmental Context and Resources | Resource (facilitator) | I suppose if we don't really know much about the patients, then to get in that collateral history from the family. So speaking to the family or like the CPN or the social workers, or the professionals that the patient that was under before they came to me, because they might hold some vital information that then would aid us to make that. Therapeutic relationship quicker. - PARTICIPANT 11 / usually try to make sure that it's the member staff that they have a good relationship with, [they] will go and do the awkward, difficult conversation. - PARTICIPANT 10 / We need interventions for staff as well, staff feeling supported staff feeling psychologically safe because I don't think carers can offer compassionate, safe care if they're feeling vulnerable or burnt out, or that they don't feel supported. - PARTICIPANT 16 / We try and get the person who knows the patient the most, even if it is a short period of time. Who's got that relationship already to try and sort of like lead on that intervention? Whoever's got the best relationship, really and even if it is like ad hoc, we do try and drive like gender and what their preferences are. – PARTICIPANT 9 / When people say, restrictive practises are always last resort and it's just not. It's not because that would mean that we're optimising every other area and we're not. It's just last resort of what we have available to us, which isn't enough - PARTICIPANT 18 / I do feel there is a lack of experienced leadership on the ground that, you know, their roles involve other things that take them away from the wards for much of the time. These ideas of psychological safety and thinking holistically about somebody's care and managing risk in an appropriate way, there's something lacking at that kind of systemic level that there's these problems that are inherent - PARTICIPANT 1 / “When we lose people in our system, you've not got 10 people in the wings biting off someone's elbows to try and get their post. That doesn't happen. Those post quite often are unfilled for a really lengthy amount of time. So with under resourcing and lack of attractiveness to people, you're trying to create change in an already really high acuity, low resourced environment that's always very stretched and very taut. It means that proactive methods are often neglected.” – PARTICIPANT 18 |
|  | Creating Safe Spaces for Patients (facilitator) | I will still people away from meeting in bed areas so that they [patients] see that they have a right for that space to feel restorative and safe, and then actually, if we're about to have conversations that might at times be emotionally charged, doing that somewhere that's more neutral might be helpful. - PARTICIPANT 16 / There's small things that make the biggest impact…we've been reminding staff to knock and wait if you want to go into their [patient] bedroom. - PARTICIPANT 19 / Sometimes people would be admitted very, very unwell and have absolutely no idea what they were doing, where they were, who they were, and they'd just be quite free and going in and out of bedrooms and taking stuff and wandering on the wards. So I used to be quite protective over that because the bedroom should be there safe space. – PARTICIPANT 6 / I've talked about in supervision is like the helpers dance and saying that it takes the system, the person administering the intervention and the patient to collaborate and I'd say most often, it feels that, you know, the system wants to help, we want to help but the person doesn't want to collaborate…they're just too unwell. - PARTICIPANT 14 / We work psychologically within the limitations of that restriction. Between me and the rest of the AHPs we have 11 different groups or activities that we facilitate each week to try and continue to promote that psychological safety on the ward, even with the restrictions. - PARTICIPANT 17 / “I've been working with a lady who doesn't want males to touch her. So that's her plan. If we get to a point where she's really distressed and she's trying to assault staff, we have to make sure it's a female team… I think trying to be trauma aware in that way in terms of getting her feedback and listening to her and listening to her family. - PARTICIPANT 5 / They have the right to make their own unwise decisions. - PARTICIPANT 17 / We try to remain respectful and give people the choice if they want to be involved or not and have as little or as big part in [that decision] as they want. – PARTICIPANT 2 |
|  | Ward Environment (barrier) | If we see somebody who's a self-harmer, who might have a EUPD and then we've got a really prolific self-harmer on the ward, they will trigger that person who's got EUPD to then have a relapse in their emotional journey. - PARTICIPANT 3 / When you're in inpatient services, people don't often build relationships with each other. They kind of just, like, coexist, don't they? - PARTICIPANT 2 / The whole dynamics of the ward can change with the addition of one patient. – PARTICIPANT 6 There's a lot of restrictive practices in terms of bedrooms being locked between certain time periods during the day. The kind of rationale for that sometimes is to avoid incidents. - PARTICIPANT 13 / I think the wards where people would just be like, well, this is how it is. And that's how it's done. And that's how we've always done it had the highest level of incidents and were psychologically unsafe. - PARTICIPANT 6 / I think the only thing I would change, which patients often say about is that because we have a mixture of formal and informal patients, the front door obviously has to be locked. I don't know how they would ever change that, but informal patients say, well, I can come and go as I want, you know, and I can't even get out the front door without having to be asked to be let out. - PARTICIPANT 9 / I think that mental health system as a whole probably doesn't support psychologically safety. Our environments are a huge [one]. - PARTICIPANT 5 / I think it's definitely something to aspire to, but I can see how inpatient mental health might fall short of that in certain situations. - PARTICIPANT 12 / No one in their right mind would go into this job if you didn't have a real passion and a real motivation to provide good quality care for the most traumatised individuals of society. I do truly believe in that. These jobs are not glamorous, they’re minging. They're really awful pay in awful environments with really high acuity. It is grossly unpleasant. - PARTICIPANT 18 |
|  | Lack of time (barrier) | What's going to take up more of my time and resources giving an IM or sitting and having to chat with someone to deescalate them for half an hour to an hour? - PARTICIPANT 17 / To get people to be psychologically safe takes a lot longer than maybe being physically safe, and it feels like we've not got the time to sort of achieve that. - PARTICIPANT 15 / People don’t have the time or the resources to talk to people for any length of time to actively engage people so much. - PARTICIPANT 1 |
|  | Staff Culture (barrier) | If systemically staff are always working in a culture of threat and feeling unsupported, then I feel that that drains their ability to offer that well-rounded care. You know, the core nursing team on shift most times are within two years of qualification. So for whatever reason a systems level, there's something happening where staff retention is having a real impact. - PARTICIPANT 16 / I think it's difficult to feel physically safe on the ward sometimes. I know sometimes psychiatrists use that as a question in ward reviews of like, do you feel safe on the ward to kind of probably more to inquire about kind of like feelings of psychosis and paranoia. But if someone says no, I kind of think well, no, I wouldn't either so. - PARTICIPANT 15 / We just don't have a lot of experience in working with people with mental health problems and they learn from other staff around them who probably also don't have a lot of experience and it just becomes this really negative culture of this is how we respond. - PARTICIPANT 13 / “I think on reflection probably the environments that I was working in in those situations were probably a little bit toxic. And psychological safety wasn't kind of like high on the agenda.” - Participant 2 |
| Beliefs About Capabilities | Importance of Understanding Actual Risk (facilitator) | Lots of these restrictive practises are agreed with people and they understand the rationale for it. Sometimes it's not, but when it is, I think that's maybe when you get that psychological safety, when there's been a time when you can discuss those practises, when someone's not in distress. I think those conversations around restrictive practises are what helps people to feel psychologically safe. – PARTICIPANT 19 / This individual had a sock that they were pulling round their neck and making all sorts of gurgling noises and everything. I saw it wasn't tied but the young nurse was panicking, so I just sat down with the person. I said, what's going on? I can see you're really, really distressed. It's not going to work, but I recognise you're not having a really nice time here. What's happening for you? Is there anything that we can do here now? And it worked nobody had to lay a finger. - PARTICIPANT 20 / We have to risk assess each patient daily to make sure that the level of risk that they present is sort of counteracted in what we allow them to do on the ward as, as a free person, as an individual who's got autonomy over their own decisions. - PARTICIPANT 3 |
|  | Dealing with Complexity (facilitator) | So we want to move away from challenging behaviours because that feels quite blaming and that and actually we know that lots of our environments are challenging, which then in turn, you know people become distressed. - PARTICIPANT 5 / Some one patient is going to want to talk about it two hours after they've woken up. Another person isn’t ready to reflect until the next day or the day after, or even the week after. But at the time your reflections are different from what they are later so. I think that comes back around to sort of you know, when they are leaving we go out and have discussion around how not to come back here again in the nicest possible way. - PARTICIPANT 10 |
|  | Expectations vs Reality (barrier) | I think some people think that going to come to hospital, they're going to get a therapy every day and that it's going to be this marvellous kind of magic, onetime thing and that’s not what it's like. That's not what we're able to do. I'd love to be able to do that. But we can't, and it's sort of managing those expectations around that. - PARTICIPANT 15 / I cannot make a massive difference like the sole recovery of this patient is not dependent on me. Like it's nice if you can, like offer them support and they can have like a trusting relationship with members of staff. But I can't save the world here like I can't make this better. Like you can't just have to get on with your job and just sort of do it. - PARTICIPANT 12 / There’s pockets of opportunity where we will tap in and try make it therapeutic but at other times the individuals got a complete stranger looking over them you know. - PARTICIPANT 8 |
|  | Conflicting Priorities Limiting the Possibility of Psychologically Safe Care (barrier) | Our ward tries to stick to it quite closely to have 72-hour admissions for BPD diagnoses. But recently we really advocated and said I know this person has an EUPD diagnosis, but she's had a lot of acute stresses recently and would benefit from being in this environment that's safe for her for a little bit of a rest, a little bit of time to feel contained. We can't just discharge her yet. - PARTICIPANT 14 / I think it would my gut reaction is, no management and leadership do not prioritize psychological safety. It's that physical safety that's the priority. - PARTICIPANT 4 / I think probably the people you get on acute wards are different now to what they were then. I think they’re more desperate so I wonder if it’s actually got slightly worse because of the press pressure to get people out more quickly. Just patched up. - PARTICIPANT 1 / It seems inhumane to lock people out of their rooms all day. Even if you are saying it's for a certain reason, when you try and find out the reason behind it, it's often all we don't want incidents to happen, so they'll lock someone out of their room between the hours of nine and five. - PARTICIPANT 13 / I think sometimes we're a bit like too well-oiled of a machine with it and it's sometimes you know, we’re the people that have to say, hang on, we need to think about this person, although they've got this diagnosis and this history, let's think about it again. - PARTICIPANT 14 |
| Knowledge | Healthcare Professional – Patient Relationship (facilitator) | Rather than getting het up on what they said, knowing them well enough to know that that is an indication of illness, and actually, if he's started speaking like that again, we need to do something about the treatment plan, because that's that's a sign of his illness - PARTICIPANT 10 / We ask them to complete a Safewards plan. So it's asking them like what they like, what they don’t like, how do they like to be approached? And any sort of like specific gender that they prefer to work with. It ranges from absolutely everything, do they prefer a room with a toilet or without a toilet, just sort of things like that. - PARTICIPANT 9 / But when they're engaging in self harm, communicating with them, giving that autonomy, not just going straight in like I did in my first year in the NHS of someone head banging, just going in and restraining them, and then it just turning into a brawl. - PARTICIPANT 17 / If anything, it's more beneficial to be providing that sort of in depth, bespoke, sort of therapeutic interventions. Otherwise, people tend to get stuck and then it's sort of like prolonged in terms of the amount of time that they spend in segregation and also just in general by a person being put into inpatient mental health services and then being admitted for a stay. - PARTICIPANT 2 |
|  | Knowledge Gaps (barrier) | Quite a lot of misunderstanding from staff. A lot of the time [they think] that patients are just being difficult because they don't want to eat or they’re just acting up and that it’s just behavioural - PARTICIPANT 13 / You could put them on every training course in the world, but unless they sort of see certain things first hand and experience certain things first hand. Say for example, working in a trauma informed way, psychological safe way they have to understand actually what that means in practice. - PARTICIPANT 2 / “It leaves me with a kind of sense of anxiety that I'm missing something and not supporting my team as much- you know I'm missing a key element or key understanding about it [psychological safety].” - PARTICIPANT 4 |
| Social/Professional Role and Identity | Professional Confidence to Make and Explain Decisions (facilitator) | We've been trying all the methods of communication with her as well. So we use like symbol based communication we’ve been trialling that with her instead so using other avenues of people to communicate or. Trying different things as well, so continually making sure that offer is there reminding them of that or trying to support them with like basic needs on the ward and having a dialogue about that. - PARTICIPANT 6 / I mean, we have done a lot of things on a PICU that people would be like you absolutely should not have done that, that was way too risky. But I know because of the therapeutic relationships that I build with patients that I can risk assess that. - PARTICIPANT 11 / I think in terms of creating that culture change. One person acting differently could have sort of like broken that cycle a little bit, so I think. Now I confidently speak out and professionally challenge things. - PARTICIPANT 2 |
|  | Staff Can Add Value to Ward Processes (facilitator) | If you're talking more than half the time, then you're talking too much. Shut up. Listen to the patient and find a way of communicating the bits of information you want to get across to the patient through a question rather than through direct information. - PARTICIPANT 4 / What helps you to get alongside somebody and think together about what's going on for you in a way that feels contained and safe and not overwhelming and intrusive. - PARTICIPANT 19 / Her key worker, who's our main nurse on the ward, she never gets involved with any restraint and any feeds because actually, we felt that that would really impact on her relationship with her. So she feels quite psychologically safe with [name removed] the nurse that that is her key worker. - PARTICIPANT 7 / She was the activity coordinator, so her point was to make nice things for the patients to do between their therapy. [She’d say] “We're having a pamper session next, I'd love to go out and pay £50 to get this done, but sure, come on in the NHS will fund it”. The patients picked up on her incredibly cynical approach and then she'd be like, “well, they don't come to the groups anyway”. And I'm like, I wonder why! - PARTICIPANT 17 |
|  | Inconsistency in Ways of Working (barrier) | It’s management dependent but I do think that everyone tries in their own way, but without the proper definition or training, then everyone's doing their own concept of it. - PARTICIPANT 6 / She had every skill under the sun and when she'd escalate, the staff would resort to either this PRN or we're going to IM you instead of encouraging her to use any of the skills she'd learned over 18 weeks of DBT. I don't tend to work outside the hours of 8:00 AM till 6:00 PM. And you'd often find that escalations happened after 6:00 PM when we weren’t on the ward to sort of influence or support with the situation…it would just be straight to PRN or straight to IM. - PARTICIPANT 17 / When people don't stick to boundaries you hear patients say, oh, but last night, so and so said I could do whatever. Inconsistent use of boundaries is really unhelpful because that’s really difficult to then implement the same boundary the next time. - PARTICIPANT 10 / It’s really hard to kind of remain consistent in that care and they say that is what is most beneficial, like especially for people like that might have autistic spectrum conditions you need consistency. They need to know what to expect. They need to know what's coming at them. So I think it's very difficult to kind of feel like you're doing your job and feel like you're doing your best for the patient when you're getting conflicting things all the time. - PARTICIPANT 12 |
| Social Influences | Collaboration and Shared Priorities (facilitator) | I think you know you need people,staff team doing the day-to-day work who are fully bought into these ideas and may be accountable for putting these ideas into practise or not. - PARTICIPANT 16 / The meetings that we have are just really great because it shows that we are looking at how we can reduce that [restrictive practices] every month. I think collectively it's great for different professionals from different backgrounds to go and different age ranges and [people from] different services because it gives you the opportunity to share ideas and you pick up a lot from there like oh, that sounds good. We're going to do that. - PARTICIPANT 9 |
|  | Staff vs Patients: Preventing Engagement with Care (barrier) | Sometimes we find that when somebody is on a one to one or a two to one, it will trigger somebody else because they're not getting that same attention, so have to put something in place to obviously make sure that we're not then going to end up with two patients on a two to one because of their triggers and emotions. - PARTICIPANT 3 / So we're like, it's more or less like punishing them for having an incident, which people then will say like, oh, they need to take responsibility. There needs to be like a consequence, but like we're actually in a hospital and we're looking after people. - PARTICIPANT 11 |
|  | Resistance to Change (barrier) | Some staff that see psychological safety as a kind of a woolly and hippie idea, I think, because it's not what they've always delivered, they will be the knuckle draggers, that kind of the last to adapt to any of that makes sense. - PARTICIPANT 4 This- psychological safety is seen as extra stuff but it isn't, this should be absolute bread and butter. So I'm a little bit of a stickler, so when I hear kind of management say well if we can't deliver on this, we can't do the bells and whistles and I'm like this stuff isn't bells and whistles pet like this stuff is like is what you should be delivering. It should be non-negotiable. - PARTICIPANT 18 / They listened to us a lot more and I don't know whether that's also because we've changed consultants. I don't know whether that's a part of it. And different views of different consultants and that can have a big impact on that as well. – PARTICIPANT 14 / I have very little autonomy on the ward. You know, I might well try and persuade the consultant to think about a different treatment plan. But if he's not going to go with that, I have no autonomy. I can't undermine the consultant, I know I just need to accept that on the chin and move on. - PARTICIPANT 4 / Especially as a loan psychologist in those settings you're pushing back against a culture that is really quite opposite to what you're trying to trying to embody and what you're trying to offer. - PARTICIPANT 16 / I do find it is very consultant or psychiatrist dependent on how psychology is received on the ward and how driven the medical model is in comparison to any other models of care. - PARTICIPANT 17 |
| Emotions | Empathy and Compassion (facilitator) | It's just about seeing the person in front of you. Seeing a person, not a diagnosis. Asking the question what happened rather than what's wrong. - PARTICIPANT 20 / I can't imagine being a patient on a ward and already feeling. So ill and so confused and and then having these awful things happen like it's just heartbreaking. You know it's more intrusive sometimes than the mental illness. I think the level of scrutiny that you're under on the wards and everything that you do is written about, whether you like it or not. - PARTICIPANT 6 / You wouldn’t go to someone in distress on the street here have a lorazepam. You just wouldn't. You'd talk to them. You'd sit with them. If your daughter was having a tantrum, you wouldn't be like quick Calpol like it just wouldn't happen. - PARTICIPANT 17 / You can keep her at home and look after her. Just keep her at home and I said do not let her go into that system because she'll just be bouncing around for years. - PARTICIPANT 6 / “I don't think they [colleagues and organisations] recognize the impact that just being in an inpatient unit has on a person. It's almost like the impact of being around so many other people who are unwell, is forgotten...there just isn't that understanding that actually a person can go into an inpatient unit with no trauma and come out with a shed load just because they've been in that place.” - PARTICIPANT 13 |
|  | Burnout and Compassion fatigue (barrier) | Like it'll be really acute, and staff will be really burnt out. Definitely sort of psychological safety will go down like just in effort to sort of like get through the day almost like, get everything done that needs to be done. -PARTICIPANT 12 / I think you see a lot of compassion fatigue. And you see a lot of this towards patients who are frequent users of healthcare - PARTICIPANT 6 / We are in the middle of a colossal staffing crisis at the moment in mental health services. I think while we have made gains up to about 12 years ago, I really worry about where we're at at the moment because staff are exhausted. - PARTICIPANT 4 / But those nursing assistants, are the ones doing the observations, and at the very minimum, we'll have a patient on an hourly observation and the very most would have them one to ones. But if you've got a 10 minute observation and you're constantly going around checking on an individual and you find that they've ligatured and they're blue and you kind of kind of set your alarm off and then work on that individual. And you know, you spend some time with that individual. And then 20 minutes later, you go and do your check and find exactly the same situation. And over the course of your shift it's going to have a psychological impact and it is going to increase burnout, you know and as someone that has to manage colleagues with sickness and absence have definitely seen a spike on that. - PARTICIPANT 8 |
|  | Fear and Personal Opinions Driving Decisions About Patient Care (barrier) | I can't bring that person out of seclusion because if something happens, I'm going to be told off. I can't reduce that person's observation levels because God forbid something happens. It's on my head. So that then and for me, they are the individuals who don't feel like they've got psychological safety because it's that blame if something goes wrong. - PARTICIPANT 5 / The priority is saving everyone's skin, saving everyone's reputation and not wanting to take positive risks because of the potential. - PARTICIPANT 13 / you did see staff members that probably did enjoy it more than they should. Maybe that patient had annoyed them or they didn't like them. - PARTICIPANT 6 / Maybe like the Matron and other people who are really just push and say, no, we need that borderline out, that kind of thing. You know, because I think they just see the diagnosis. They don't necessarily look beyond that and I think we're often reminding people now it is a person. - PARTICIPANT 14 / “It feels like there's more of a concern over reputation than there is about how the patients feel because currently it feels like no matter what patients do they don't have any rights, like they're not really involved in their care. They're all sectioned.” - PARTICIPANT 13 |
